# Observation of polarity-switchable photoconductivity in III-nitride/MoS_x_ core-shell nanowires

**DOI:** 10.1038/s41377-022-00912-7

**Published:** 2022-07-19

**Authors:** Danhao Wang, Wentiao Wu, Shi Fang, Yang Kang, Xiaoning Wang, Wei Hu, Huabin Yu, Haochen Zhang, Xin Liu, Yuanmin Luo, Jr-Hau He, Lan Fu, Shibing Long, Sheng Liu, Haiding Sun

**Affiliations:** 1 School of Microelectronics, University of Science and Technology of China, Hefei, 230029 China; 2 Hefei National Laboratory for Physical Sciences at the Microscale, Department of Chemical Physics, University of Science and Technology of China, Hefei, 230029 China; 3 Department of Materials Science and Engineering, City University of Hong Kong, Kowloon, Hong Kong SAR, 999077 China; 4 Department of Electronic Materials Engineering, Research School of Physics and Engineering, The Australian National University, Canberra, ACT 2601 Australia; 5 School of Microelectronics, Wuhan University, Wuhan, 430072 China; 6 The CAS Key Laboratory of Wireless-Optical Communications, University of Science and Technology of China, Hefei, 230029 China

**Keywords:** Nanowires, Optical sensors

## Abstract

III–V semiconductor nanowires are indispensable building blocks for nanoscale electronic and optoelectronic devices. However, solely relying on their intrinsic physical and material properties sometimes limits device functionalities to meet the increasing demands in versatile and complex electronic world. By leveraging the distinctive nature of the one-dimensional geometry and large surface-to-volume ratio of the nanowires, new properties can be attained through monolithic integration of conventional nanowires with other easy-synthesized functional materials. Herein, we combine high-crystal-quality III-nitride nanowires with amorphous molybdenum sulfides (a-MoS_x_) to construct III-nitride/a-MoS_x_ core-shell nanostructures. Upon light illumination, such nanostructures exhibit striking spectrally distinctive photodetection characteristic in photoelectrochemical environment, demonstrating a negative photoresponsivity of −100.42 mA W^−1^ under 254 nm illumination, and a positive photoresponsivity of 29.5 mA W^−1^ under 365 nm illumination. Density functional theory calculations reveal that the successful surface modification of the nanowires via a-MoS_x_ decoration accelerates the reaction process at the electrolyte/nanowire interface, leading to the generation of opposite photocurrent signals under different photon illumination. Most importantly, such polarity-switchable photoconductivity can be further tuned for multiple wavelength bands photodetection by simply adjusting the surrounding environment and/or tailoring the nanowire composition, showing great promise to build light-wavelength controllable sensing devices in the future.

## Abstract (web summary)

A novel polarity-switchable photoelectrochemical photodetector based on III-nitride/MoS_x_core-shell nanowires is constructed. Such unique device architecture provides a new route for multiple-band spectrally distinctive photodetection.

## Graphical abstract

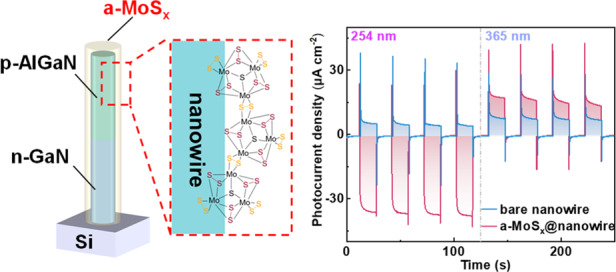

## Introduction

III–V semiconductor nanowires possess fascinating material properties, including tunable bandgaps, superior tolerance to lattice mismatch, large surface-to-volume ratio and excellent mechanical flexibility etc., which pave the way toward the fabrication of next-generation nanoscale electronic and optoelectronic devices, such as transistors^1,2^, light emitting devices (lasers^3–7^ and light emitting diodes^8,9^), photodetectors^10,11^, and solar cells^12,13^, etc. In particular, owing to their distinctive nature of one-dimensional geometry and large surface-to-volume ratio, III–V nanowire platform offers versatile strategies to manipulate the intrinsic material properties in the pursuit of novel applications while exhibiting new material-wise challenges that do not exist in their bulk form^14–16^. For instance, on one hand, such vertical and high-aspect-ratio geometry (1) enables core-shell device architecture in the radial direction which facilitates efficient light absorption and photo-generated charge-carrier extraction for high-efficient photovoltaic or photodetection applications^17,18^, and (2) provides high density of surface sites for artificial photosynthesis or photoelectrochemical photodetection^19–22^. On the other hand, ultrahigh surface areas naturally induce a high density of surface states^23,24^, which could act as charge trapping centers and harmful to the performance of nanoscale devices. Thus, effective surface passivation approaches are then required to remove or suppress such surface states to boost nanowire device performance^25^. In essence, such remarkable one-dimensional geometry provides us great opportunities to leverage their surface characteristics (for example, through surface decoration or modification by a functional layer) to spark new phenomenon which is barely attainable in conventional nanowires, paving the way for developing next-generation nanoscale electronic and optoelectronic devices.

Interestingly, amorphous molybdenum sulfide (a-MoS_x_), a family member of transition-metal chalcogenides materials, has become a rising star in the pursuit of highly efficient energy harvesting and conversion^19,26–32^. Attributing to the unique two-dimensional networks or unfolded one-dimensional chains structure of a-MoS_x_ which is bridged by disulfide ligands, abundant surface reaction sites could contact actively and closely with the surrounding environment, demonstrating brilliant reaction activities and efficient charge separation and transport^19,26–28,33,34^. More importantly, a simple electrodeposition method in room temperature could easily synthesize a-MoS_x_ material on the conductive substrates, in other words, the a-MoS_x_ can be directly coated on the nanowire surface, to enable coupling and interaction between the a-MoS_x_ and nanowire. Thereby, by leveraging the advantage of highly electrochemical active terminals of a-MoS_x_ and the ultrahigh surface-to-volume ratio of crystalline III–V nanowires, III-nitride/a-MoS_x_ core-shell nanostructures can be constructed, to not only address the surface states issue of the III-nitride nanowires, but also expand the device functionalities by unleashing the full potential of both low-dimensional materials.

In this article, we combine earth-abundant molybdenum sulfides with the popular group III-nitride semiconductor nanowires to construct a spectrally distinctive photoelectrochemical photodetector with high photoresponse and excellent tunability. Essentially, the pursuit of polarity-switchable photoconductivity behavior has recently attracted considerable interests^35–37^, because the polarity-switchable photocurrent can be employed to distinguish spectrum bands while measuring corresponding light intensity, which has been realized in many solid-state devices^37–40^. The proposed III-nitride/a-MoS_x_ core-shell nanostructures demonstrate a polarity-switchable photoconductivity under different-energy photon illumination, i.e., it exhibits a polarity-switchable photoresponse with a responsivity of −100.42 mA W^−1^ under 254 nm illumination, and 29.5 mA W^−1^ under 365 nm illumination, one of the highest value among reported polarity-switchable devices^36,38–50^. Moreover, the underlying mechanism of polarity-switchable photoconductivity behavior is revealed via density functional theory (DFT) calculations. Importantly, the responsivity and spectral tunability of the device can be further improved by simply adjusting the surrounding environment and/or tailoring the composition of the core-shell nanowires which is worth of future investigation.

## Results

### Operation of nanowire-based spectrally distinctive photoelectrochemical photodetector

**Fig. 1:**
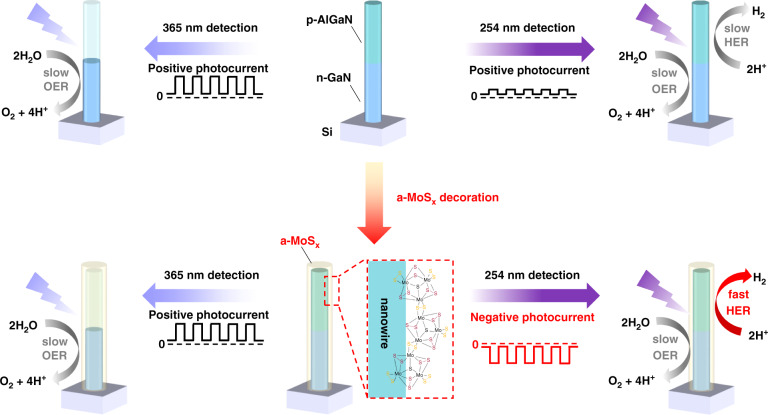
**Operation of the nanowire-based spectrally distinctive photodetector.** Schematic illustration of the nanowire structures, operation of nanowire-based photoelectrochemical photodetectors under different light illumination and the corresponding chemical reaction process at nanowire surface: Top: p-AlGaN/n-GaN nanowire; Bottom: a-MoS_x_ decorated p-AlGaN/n-GaN nanowire

Figure 1 illustrates the nanowire architectures and their simplified working principle in the photoelectrochemical environment. More detailed explanation can be found in Supporting Information. During operation, the photoresponse signal of the photoelectrochemical device is determined by the number of photo-generated carriers which effectively participate in the redox reactions, and the photocurrent polarity (either positive or negative) is determined by the type of predominate redox reaction triggered at solid/liquid interface^41^. In other words, depending on the wavelength of the incident light, either hydrogen evolution reaction (HER) or oxygen evolution reaction (OER) can dominate in the photoelectrochemical photodetector, leading to the switch of photoconductivity.

Here, our nanowires consist of 200 nm Mg-doped p-AlGaN top segment and 200 nm Si-doped n-GaN bottom segment, vertically grown on the Si substrate. The nanowires were synthesized through plasma-assisted molecular beam epitaxy (MBE) and the photoluminescence (PL) spectrum of the as-grown sample was shown in Fig. S1. The MBE-grown p-n junction based on III-nitride nanowires enables efficient light absorption and photo-generated charge-carrier extraction, more importantly, they are highly beneficial for exposing high-density catalytic sites for the PEC process, not to mention its superior crystallinity and photoelectric properties^19–21^. The schematic illustration of 365 nm photodetection process of bare p-AlGaN/n-GaN nanowires was shown in Fig. S2. Owing to the large bandgap of p-AlGaN (Fig. S1), the top p-AlGaN segment is transparent to 365 nm photons, thus photoexcitation process is absent in p-AlGaN segment and only the n-GaN segment could absorb the 365 nm photons and generate electrons and holes. According to the upward band bending of n-GaN segment in electrolyte (Fig. S3), the photogenerated holes drift toward the nanowire surface and participate in the OER (marked as process I in Fig. S2). Meanwhile, the band bending induces the electrons transfer toward the external circuit (marked as process II in Fig. S2), displaying a positive photocurrent.

Whereas, under 254 nm illumination, both the top p-AlGaN and bottom n-GaN segments could absorb the high energy photons. As shown in Fig. S4, in the p-AlGaN segment, there were two electron transport processes: the charge transport of electrons toward the surface caused by the downward band bending (process I) and the electrons drift to opposite direction caused by the build-in electric field in the p-n junction (process II). Regarding to the hole transport, the downward band bending pushes photogenerated holes in p-AlGaN segment migrate toward the space charge region (process III). The process IV indicates that the holes from p-AlGaN and the electrons from n-GaN tend to recombine within the p-n junction. It is highly possible that the existed surface states^51^, interfacial trapping states^52^, shallow trap and deep acceptor states in our nanowires^53^ may act as recombination centers for process IV. Besides, because the depletion region of our p-n junction is directly contacted with electrolyte, the corresponding surface band bending may also affect the recombination process. According to previous reports, the probability of “tunneling” becomes much larger for carriers located close to the sidewalls of the nanowires and the indirect recombination between electrons close to the core and holes close to the surface of the nanowire might be dominant^54,55^. In the n-GaN segment, on one aspect, the electrons migrate (process V), recombine (process IV) and holes drift to external circuit (process VI). On the other aspect, the existence of upward band bending at n-GaN surface not only pushes certain amount of photogenerated holes drift to the nanowire surface and participate into OER (process VII), and but also induces the electrons toward the external circuit (process VIII). Although the holes in n-GaN could also drift to space charge region induced by the build-in electric field in p-n junction (process IX), the chance is relatively small due to the existence of large band offset between the p-AlGaN and n-GaN. Essentially, since the hydrogen adsorption energies (Δ*G*_H_) of bare p-AlGaN/n-GaN nanowire surface is not suitable for efficient HER, electrons in p-AlGaN could not immediately participate in reaction once they are generated (in another word, the HER process is slow), consequently, the process II, VII, VIII determine the directionality of photocurrent rather than the process I, III, IV, V, VI. In another word, the OER process still dominates the direction of net photocurrent in bare p-AlGaN/n-GaN nanowire, resulting in a lower but still positive photocurrent under 254 nm illumination. This indicates that modification of the Δ*G*_H_ of III-nitride nanowire surface may be one of the critical factors in the pursuit of spectrally distinctive photodetection, to realize the wavelength-induced polarity-switchable photoconductivity behavior^20,41,56^.

### Structural characterization and photo-response behavior evaluation

**Fig. 2:**
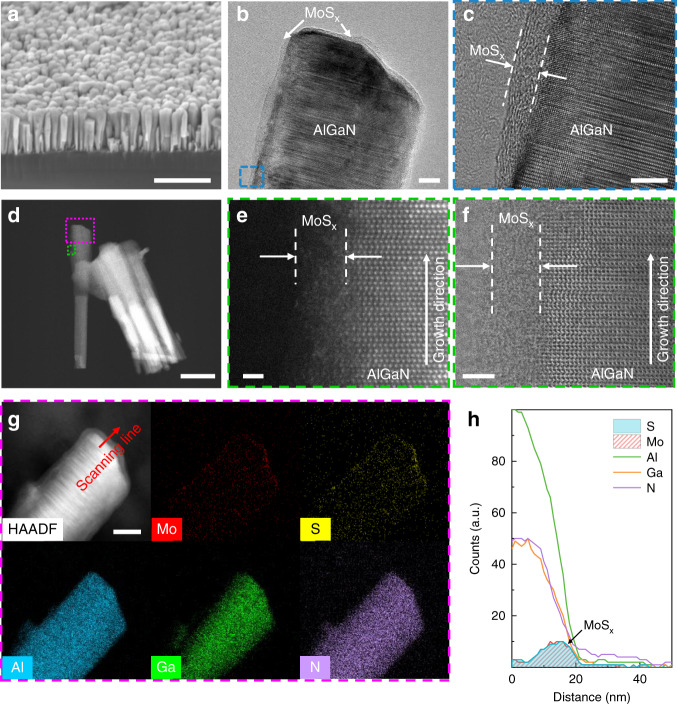
Structural characterization of the a-MoS_x_-decorated p-AlGaN/n-GaN nanowires. **a** SEM (scale bar = 500 nm), **b** low-magnification TEM (scale bar = 10 nm) and **c** high-resolution TEM images (scale bar = 5 nm), where a core-shell nanostructure can be clearly observed. The white dotted lines indicate the boundary of crystalline p-AlGaN core and amorphous MoS_x_ shell. **d** Low-magnification STEM (scale bar = 100 nm), **e** high-angle annular dark-field (HAADF) (scale bar = 1 nm), and **f** annular bright-field (ABF) STEM images of a-MoS_x_-decorated p-AlGaN/n-GaN nanowires (scale bar = 2 nm), showing the polymer nature of a-MoS_x_. **g** STEM-EDS elemental mapping (scale bar = 30 nm) and **h** line profiling showing the abruptness interface between a-MoS_x_ and p-AlGaN alloys

To achieve polarity-switchable photoconductivity under different wavelength illumination, we decorate the III-nitride nanowires with the a-MoS_x_ shell (a-MoS_x_@p-AlGaN/n-GaN) to boost the HER performance, as illustrated in Fig. 1. After a-MoS_x_ decoration, the hydrogen adsorption energies (Δ*G*_H_) at nanowire surface is expected to be modified. As a result, the electrons in the p-AlGaN segment could immediately participate in reaction once they are generated (in another word, the HER process is fast). Consequently, the process I, III, IV, V, VI may become the dominated carrier transport process rather than the process II, VII, VIII, leading to a net negative photocurrent (Fig. S5). The detailed electrodeposition process could be found in Experiment Section and Fig. S6. To further characterize the microstructure of a-MoS_x_@p-AlGaN/n-GaN nanowires, scanning electron microscope (SEM), transmission electron microscopy (TEM) as well as scanning transmission electron microscopy (STEM) and energy-dispersive X-ray spectroscopy (EDS) were performed. The SEM image of the MBE-grown p-AlGaN/n-GaN nanowires on planar n-Si (111) is shown in Fig. 2a. Figure S7 shows a representative low-magnification bright-field (BF) TEM image of nanowire arrays. The nanowires have a length of ~400 nm and a diameter of ~60 nm. As shown in Fig. 2b, a shell layer with a brighter contrast can be observed on the surface of p-AlGaN/n-GaN nanowires. As shown in Fig. 2c, in contrast to the highly crystallized AlGaN core, a ~4 nm shell layer can be observed on the nanowire surface, with no apparent lattice fringes, directly revealing the amorphous nature of the as-prepared a-MoS_x_ shell. We further employed high-angle-annular-dark-field (HAADF) STEM and BF images to characterize the structural property of a-MoS_x_@p-AlGaN/n-GaN nanowires and determine the corresponding atomic structure. Benefiting from the Z-contrast of the dark-field STEM, we notice that the n-GaN segment is much brighter than the p-AlGaN segment, as shown in Fig. 2d. The AlGaN surface is covered by a-MoS_x_ in the core/shell structure, as shown in Fig. 2e, f. The amorphous shell layer could be easily distinguished from the highly crystallized AlGaN alloys. The typical wurtzite arrangement of atoms can be assigned to the Al/Ga atoms, whereas the randomly atom layer without crystal lattice arrangement is likely to be Mo atoms which bond to the (10$\bar 1$0) surface of AlGaN. According to previous studies, the building units of amorphous molybdenum sulfides (a-MoS_x_) are identified to be [Mo_3_S_13_]^2−^ clusters which arrange in two-dimensional networks or unfolded one-dimensional chains^34,57,58^. The EDS elements mapping and line scan based on STEM demonstrate that the Mo and S atoms prefer to appear at the outer radius of the nanowires, further revealing that the hetero-nanowires are indeed decorated with a-MoS_x_ species (Figs. 2g, h and S8). All these facts confirm the successful fabrication of a uniform a-MoS_x_ shell covering the entire p-AlGaN/n-GaN nanowires.

**Fig. 3:**
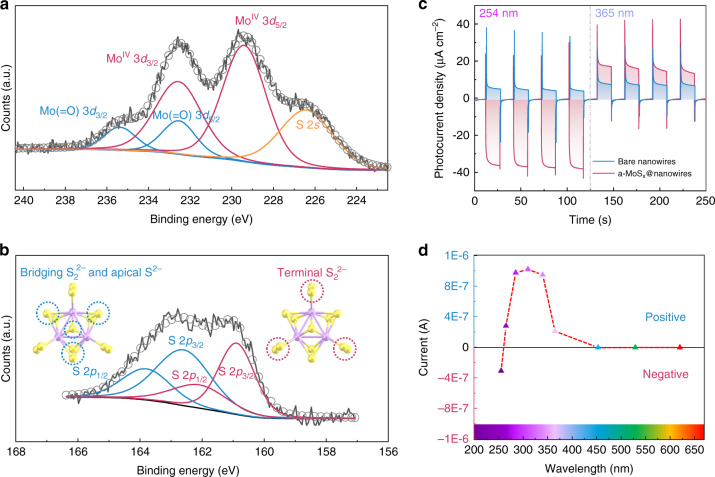
**Chemical composition and photoresponse behavior.** **a**, **b** XPS core-level spectrum and fitted peaks of S and Mo for a fresh a-MoS_x_ decorated p-AlGaN/n-GaN nanowires. **c** Photoresponse of the bare p-AlGaN/n-GaN nanowires (blue curves) and MoS_x_-decorated p-AlGaN/n-GaN nanowires (red curves) in 0.5 M H_2_SO_4_ with UV illumination at a periodic interval of 15 s at 0 V (365 nm intensity = 0.65 mW cm^−2^, 254 nm intensity = 0.4 mW cm^−2^). **d** Evolution of the current signal of a-MoS_x_@p-AlGaN/n-GaN nanowires in the wavelength range of 255–620 nm

The chemical composition and bonding configuration of the a-MoS_x_@p-AlGaN/n-GaN nanowires was further investigated by X-ray photoelectron spectroscopy (XPS). Each peak represents the binding energy between different elements in the a-MoS_x_@p-AlGaN/n-GaN structure. In Fig. 3a, the peak fitting of the Mo 3d region reveals two Mo 3d doublets and one broad S 2s peak^59^. The doublet at lower binding energies (3d_5/2_ = 229.4 eV, 3d_3/2_ = 232.5 eV) is indicative of Mo^IV^, while the doublet at higher binding energies (3d_5/2_ = 232.5 eV, 3d_3/2_ = 235.6 eV) is attributed to the Mo(=O)^34,57^. Interestingly, a broad S 2s peak can also be observed in Mo 3d region, suggesting the exist of multiple chemical states of sulfur species, which is further verified by S 2p XPS spectrum. As shown in Fig. 3b, the broad S 2p peak could be fitted with two distinct doublets: one doublet at lower binding energy (2p_3/2_ = 161 eV, 2p_1/2_ = 162.2 eV), which represents terminal S_2_^2−^ ligands, and one doublet at higher binding energy (2p_3/2_ = 162.6 eV, 2p_1/2_ = 163.8 eV), which can be assigned to the bridging S_2_^2−^ and apical S^[2– 29,60^. The Mo:S ratio extracted from the XPS spectra is 3:12.5, which is close to Mo_3_S_13_ clusters (Mo:S = 3:13) and is in agreement with previous XPS studies of electrodeposited amorphous Mo_3_S_13_^33,34^. Besides, the atomic ratio of bridging S_2_^2−^/apical S^2−^ and terminal S_2_^2−^ is equal to 6.96:6, which is also close to the structure of Mo_3_S_13_ clusters (7:6). In fact, these numbers are in good agreement with the following studies of electrodeposited amorphous Mo_3_S_13_^58,60,61^. All of the results directly corroborate the successful decoration of a-MoS_x_ on p-AlGaN/n-GaN nanowires.

To evaluate their photoresponse behavior, the constructed nanowire-based photoelectrochemical photodetectors were tested under 254 nm/365 nm illumination. The images of three electrodes architecture and photoelectrode could be found in Fig. S9. Under modulated light, both the bare p-AlGaN/n-GaN nanowires and a-MoS_x_@p-AlGaN/n-GaN nanowires show stable and reproducible on/off cycles (Fig. 3c). The bare p-AlGaN/n-GaN nanowires show positive photoresponse under either 254 or 365 nm light illumination, which is consistent with the proposed operation principle of the bare p-AlGaN/n-GaN nanowires (Fig. 1). Indeed, under 365 nm illumination, photons can only be absorbed by the n-GaN segment, triggering OER at n-GaN/electrolyte interface and thus demostrate a positive photoresponse (~8 μA cm^−2^). When the illumination was turned to the 254 nm light, due to the non-ideal hydrogen adsorption energy (Δ*G*_H_) of p-AlGaN surface, the HER process was not strong enough to reverse the polarity of photoresponse in the entire nanowires, leading to an overall smaller but positive photocurrent at 254 nm (~5.4 μA cm^−2^). On the contrary, the photocurrent signals can be reversibly switched once the nanowires are covered by a-MoS_x_ layer. As clearly shown in Fig. 3c, under 254 nm illumination, the a-MoS_x_@p-AlGaN/n-GaN nanowires exhibit a strong negative photoresponse (−38 μA cm^−2^), confirming the dominance of HER reaction at the semiconductor/a-MoS_x_/electrolyte interface. More importantly, the a-MoS_x_ significantly improves the charge transfer properties at the nanowire/electrolyte interface compared with bare nanowires, as verified by the electrochemical impedance spectroscopy analysis (Fig. S10), which led to a higher positive photoresponse (~18.5 μA cm^−2^) under 365 nm illumination. Notably, there are transient photocurrent spikes in both current-time response curves. This phenomenon can be explained as follows: the materials with wurtzite crystal structure, including the group III-nitride nanowires have strong pyroelectric properties^62^, the observed peaks in our device might be accounted for the pyroelectricity effect in the p-AlGaN/n-GaN nanowires. However, such transient photocurrent peaks can be also observed in many other non-pyroelectric systems, which were accounted for the transient carrier accumulation and recombination near the semiconductor/electrolyte interface^41,63,64^. In short, we suspect that these factors could act together and lead to the generation of transient photocurrent peaks.

With such distinctive photoresponse to the incident photons with different energies, the a-MoS_x_@p-AlGaN/n-GaN nanowire device is then able to distinguish different spectral bands by exhibiting different polarity of the photocurrent. As demonstrated in Fig. 3d, the incident light from different wavelengths of LEDs, including 255, 265, 285, 310, 340, 365, 453, 529 and 620 nm, were selected to measure the photoresponse of a-MoS_x_@p-AlGaN/n-GaN nanowires in 0.5 M H_2_SO_4_ at an applied potential of 0 V. The output photocurrent signal is negative under 255 nm light illumination, which is then switched to positive when the wavelength goes beyond 265 nm, confirming the spectrally distinctive photodetection behavior. Besides, the photoresponse to the visible light illumination is negligible, indicating the excellent visible-blind characteristic of our device.

### Tunability of nanowire-based spectrally distinctive photoelectrochemical photodetector

**Fig. 4:**
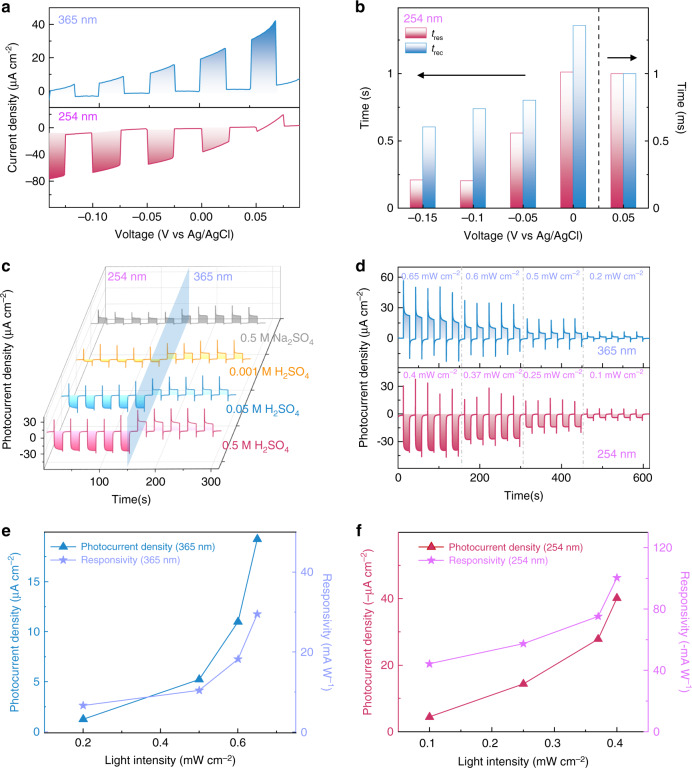
**Spectrally distinctive photodetection.** **a** LSV curves of a-MoS_x_@p-AlGaN/n-GaN nanowires with potential varied from 0.1 to −0.15 V in 0.5 M H_2_SO_4_. Scan rate is 2.5 mV/s. **b** The bias dependent photoresponse behavior of a-MoSx@p-AlGaN/n-GaN nanowires at 254 nm illumination. **c** *I*_photo_−*t* characteristics of a-MoS_x_@p-AlGaN/n-GaN nanowires in different concentration of H_2_SO_4_ (0.001, 0.05, 0.5 M) and 0.5 M Na_2_SO_4_ at 0 V. **d** Photoresponse behavior of the a-MoS_x_@p-AlGaN/n-GaN nanowires photodetector under 365 nm (top) and 254 nm (bottom) wavelength light illumination with different light intensities. The photocurrent density and corresponding calculated photoresponsivity of a-MoS_x_@p-AlGaN/n-GaN nanowires under various power intensities of **e** 365 nm and **f** 254 nm illumination. The photocurrent density and responsivity of 365 nm is positive, while the photocurrent density and responsivity of 254 nm is negative

Furthermore, the intensity of the photocurrent in a photodetector represents the quantity of photogenerated carriers that effectively participate in redox reactions. A few key parameters may have significant impact on the photodetection performance. We firstly carried out the voltage-dependent photoresponse measurements of the a-MoS_x_@p-AlGaN/n-GaN nanowires in 0.5 M H_2_SO_4_. As shown in Fig. 4a, the photocurrent density is tunable and shows great bias dependent behavior. It is noted that the photocurrent increases with varied applied bias ranging from 0.1 to −0.15 V under 254 nm illumination. The external potential could efficiently facilitate photocarrier separation and transport, and provide external voltage for HER, further boosting the photodetection process. On the other hand, the value of photocurrent under 365 nm light shows an opposite tendency with the increase of reverse bias, which can be explained by the suppression of OER. In addition to the photocurrent signal, the response speed is also an important parameter of a photodetector. Figure 4b shows the relationship between the external bias potentials and the response time of a-MoS_x_@p-AlGaN/n-GaN nanowires for 254 nm detection. The raise time is denoted as *t*_res_, while the recovery time is denoted as *t*_rec_, which represent the time required for the photocurrent to increase from 10% to 90% of the maximum value, and recovers from 90% to 10% of the maximum value, respectively. As expected, the response characteristic (both the raise time and the recovery time) is improved when the bias potential varies from 0 to −0.15 V. It is worth pointing out that, when the polarity of photocurrent changed from negative to positive, the response time dramatically decreases by an order of magnitude: from second (s) level to millisecond (~ms) level, which further verifies the switch of the photoelectrochemical process (HER vs. OER) of our PEC device, as schematically illustrated in Fig. 1 and elaborated in details in Figs. S2–5. Besides, under 365 nm illumination, because the polarity of photocurrent is positive, the response time maintains at ~ms level (Fig. S12). This phenomenon could be explained as follows: when the positive photocurrent is generated, the dominant reaction in the nanowires is OER, and the whole circuit does not require sophisticated carrier recombination process to complete the current loop, and thus exhibiting a fast characteristic. In contrast, when HER process dominates the entire photoelectrochemical process, photogenerated electrons of p-AlGaN drift to p-AlGaN/electrolyte interface to participate HER while the photogenerated holes of n-GaN drift to external circuit, leading to a negative photocurrent. As illustrated by the bandgap diagram of our nanowires (Fig. S5), because, both the n-GaN and p-AlGaN segment absorb the 254 nm light in p-AlGaN/n-GaN nanowires, the entire HER process may involve carrier diffusion, recombination, and drifting process which possibly reduce the response speed during the photodetection process, unlike the OER process where a relative faster speed can be expected because the process simply involves with the n-GaN segment under 365 nm illumination. Those observations indicate that the applied external potential is an effective method to manipulate the photodetection behavior of a-MoS_x_@p-AlGaN/n-GaN nanowires.

Furthermore, the amount of H^+^ in electrolyte also plays a significant role during the photoelectrochemical photodetection process. On one hand, the increased concentration of the conductive ions effectively optimizes the interfacial resistance, mass transfer process and the conductivity in electrolyte. On the other hand, the increased content of reactants contributes to a more effective HER reaction. Therefore, as displayed in Fig. 4c, under 254 nm illumination, when the concentration of H_2_SO_4_ increases from 0.001 to 0.5 M, the photocurrent shows an increase trend, steadily growing from −5.3 to −37 μA cm^−2^, demonstrating an electrolyte-dependent photoresponse behavior; whereas the value of photocurrent under 365 nm illumination remains at a constant level. Particularly, when replacing the H^+^ in electrolyte as Na^+^ (using 0.5 M Na_2_SO_4_ as electrolyte), the a-MoS_x_@p-AlGaN/n-GaN nanowires exhibit positive photoresponse under 254 nm light illumination due to a much reduced HER activity, implying that the polarity of photocurrent can also be effectively modulated by varying the type of electrolytes. The impact of electrolyte on photodetection process can be further confirmed by the response time measurements (Fig. S13).

Apart from the above-mentioned factors, the incident light intensity may also have a large impact on the photocurrent signals. It can be found in Fig. 4d, the photocurrent intensity of a-MoS_x_@p-AlGaN/n-GaN nanowires exhibits an obvious increasing trend upon raised incident light intensity. A significant enhancement of the photocurrent can be realized when the 254 nm light intensity is ramped up from 0.1 to 0.4 mW cm^−2^. Besides, the absolute value of the output photocurrent increases when the light intensity increases from to 0.2 to 0.65 mW cm^−2^ under 365 illumination. To better compare the output photocurrent, a critical parameter of the photodetector: the responsivity *R* is extracted from the following equation:

$$R = I_{{{{\mathrm{photo}}}}}/J_{{{{\mathrm{light}}}}}$$

where *I*_photo_ represents the photocurrent density (μA cm^−2^) and *J*_light_ corresponds to the incident light power intensity (mW cm^−2^)^65,66^. It can be learnt that both the *R* and *I*_photo_ under 254 and 365 nm light shows an increasing trend as the light intensity increases, as exhibited in Fig. 4e, f, offering a promising approach for spectrum band distinction and light intensity quantification. The calculated negative photoresponsivity under 254 nm is −100.42 mA W^−1^, and positive photoresponsivity under 365 nm illumination is 29.5 mA W^−1^, these values are one of the top among the reported spectrally distinctive photosensors, as shown in Table 1. Besides, the morphologies of nanowires before/after PEC measurement indicate that the degree of surface corrosion of our nanowires is nearly negligible after 30 min test (Fig. S15). In short, all above photoresponse results undoubtedly prove the excellent tunability of a-MoS_x_@p-AlGaN/n-GaN nanowires for versatile spectrally-distinctive photodetection in the future.

**Table 1:** Performance comparison of a-MoS_x_@p-AlGaN/n-GaN nanowires photodetector with other reported polarity-switchable devices.

Photodetector	Type	Working mechanism	Wavelength [nm]	Photocurrent magnitude	Responsivity [mA W^−1^]	Ref.
a-MoS_x_@p-AlGaN/n-GaN	PEC PD	p-n junction/PEC effect	254	μA cm^−2^	−100.42	This work
			365	μA cm^−2^	29.5	
Pt/p-AlGaN/n-GaN	PEC PD	p-n junction/PEC effect	254	μA cm^−2^	−175	^41^
			365	μA cm^−2^	31	
AlGaN/Pt-GaN cell	PEC PD	Photovoltage-competing/PEC effect	254	μA cm^−2^	11.39	^42^
			365	μA cm^−2^	−0.3	
Pt/p-GaN	PEC PD	Carrier transport/PEC effect	285	μA cm^−2^	−7.2	^43^
			365	μA cm^−2^	1.1	
α-Ga_2_O_3_/Cu_2_O	PEC PD	p-n junction/PEC effect	254	μA	0.42	^44^
			365	μA	−0.57	
Au/TiO_2_	PEC PD	Plasmon/PEC effect	400	nA	−0.6	^36^
			800	nA	0.15	
p-SnS/p-Si	Solid-state PD	Photovoltage	400	μA	−34.45	^45^
			800	μA	11.44	
Ga_2_O_3_/GaN	Solid-state PD	Photovoltage	254	nA	43.9	^38^
			365	nA	−35.8	
ZnO/Sb_2_Se_3_	Solid-state PD	Photothermoelectric/Photovoltage	400	μA	−0.0037	^40^
			800	μA	0.0145	
SnS_x_/TiO_2_	Solid-state PD	Photovoltage/pyro-phototronic effect	375	μA	1.64	^46^
			400	μA	−6.5	
ZnO/SnS	Solid-state PD	Pyroelectric/Photovoltage	365	nA	−0.155	^39^
			690	nA	0.364	
Ag/β-Ga_2_O_3_	Solid-state PD	Plasmon/Photovoltage	254	nA	0.157	^47^
			365	nA	−0.353	
MoS_2_/GaN/Si	Solid-state PD	Photovoltage	390	nA	35	^48^
			995	μA	−2.38 × 10^4^	
Sb_2_Se_3_/ZnO	Solid-state PD	Photovoltage	520	μA	−11	^49^
			905	μA	78	
SnS_2_/PbS	Solid-state PD	Photovoltage	UV	nA	10^8^	^50^
			VR	nA	10^8^	

### Theoretical calculations

**Fig. 5:**
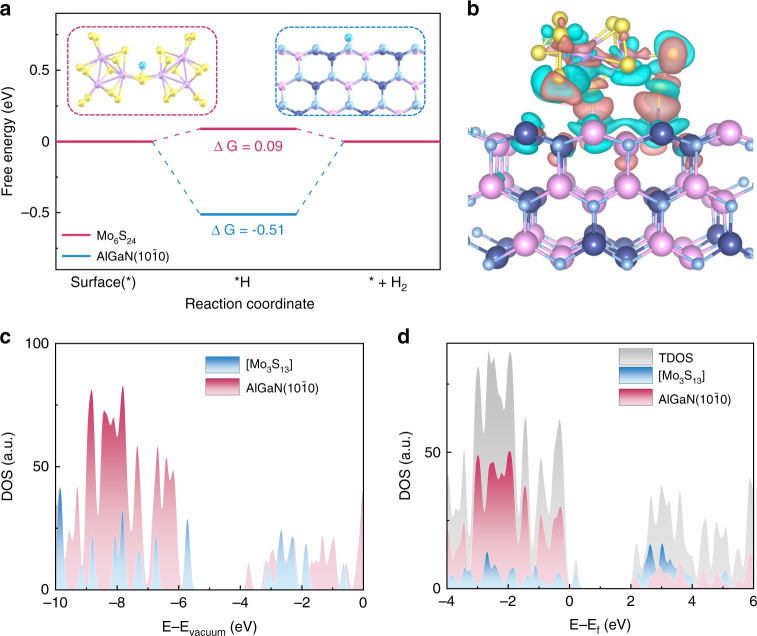
**DFT calculations.** **a** Free energy diagram for HER on Mo_6_S_24_ (red line) and AlGaN (10$\bar 1$0) (blue line). Inner figures show the configurations of *H on the Mo_6_S_24_ (red box) and AlGaN (10$\bar 1$0) (blue box). The balls in yellow, purple, sky blue, light blue, navy and pink represent S, Mo, H, N, Al and Ga atoms, respectively. **b** Electron density difference plot of the Mo_3_S_13_/AlGaN(10$\bar 1$0), the deletion and accumulation of electrons are represented by the blue and red regions, respectively. **c** The density of states of the clean AlGaN(10$\bar 1$0), clean Mo_3_S_13_ and **d** total density of states (TDOS) of Mo_3_S_13_/AlGaN(10$\bar 1$0) and the projected density of states of Mo_3_S_13_ and AlGaN (10$\bar 1$0) in Mo_3_S_13_/AlGaN (10$\bar 1$0). The energy level is relative to vacuum level in **c** and Fermi level in **d**

To gain further insights into the mechanism that how the surface modification by coating a-MoS_x_ layer influence the photodetection performance, theoretical investigations based on DFT calculation were carried out. Considering the fact that a-MoS_x_ is composed of 1D Mo_3_S_13_ cluster links^34^ and the Mo_6_S_24_ cluster is the dimer of Mo_3_S_13_ cluster which could exhibit more polymer properties, thus, we used the Mo_6_S_24_ clusters for the calculation HER and OER reaction. We first simulated the HER at the equilibrium potential with standard computational hydrogen evolution model (more details could be found in DFT calculation part). As shown in Fig. 5a, the H adsorption sites in Mo_6_S_24_ is the bridging S_2_, which is similar to the results of Mo_2_S_12_ calculated by Wu et al.^67^. It is reasonable considering the difference in coordination number and electronegativity of Mo and S, which imply the reliability of our calculations. The calculated free energy change (Δ*G*) for Mo_6_S_24_ was found to be 0.09 eV, which suggests that MoS_x_ materials are extremely suitable for HER, as the Δ*G* of an ideal HER catalyst is zero. In contrast, the calculated Δ*G* of AlGaN($10\bar 10$) is −0.51 eV, which suggests strong adsorptions of hydrogen on the AlGaN($10\bar 10$) surface and means the HER activities of AlGaN(10$\bar 1$0) surface are quite low. Hence, we can conclude that when we propose 254 nm photodetection, MoS_x_ decorated nanowires have much better HER activities than bare nanowires, causing the reverse of the photocurrent from positive to negative. At the same time, we also calculate a four-step of the OER process, which can be seen in the Supporting Information Fig. S16. To study the states change after placing Mo_3_S_13_ cluster on AlGaN(10$\bar 1$0), we calculated the binding energy by:

$$E_{{{\mathrm{b}}}} = E({\rm{AlGaN}} - {\rm{Mo}}_{3}{\rm{S}}_{13}) - E({\rm{AlGaN}}) - E({\rm{Mo}}_{3}{\rm{S}}_{13})$$

where these three energies correspond to the energy of Mo_3_S_13_/AlGaN($10\bar 10$), clean AlGaN($10\bar 10$), clean Mo_3_S_13_ cluster. The binding energy is −2.72 eV which manifests strong interaction between AlGaN and Mo_3_S_13_ cluster. To study the change of electronic state after placing Mo_3_S_13_ cluster on AlGaN($10\bar 10$), we calculated the charge density difference which is shown in Fig. 5b. It is clearly shown that the charge reduction near the S and Ga, S and Al, and the charge accumulation at the interface, indicating the strong interlayer interaction which would make it easier for electrons to transport between AlGaN and Mo_3_S_13_.

Besides, to reveal the internal mechanism of the switch of photocurrent directionality, we calculated the electronic properties of clean AlGaN (10$\bar 1$0) surface, clean Mo_3_S_13_ cluster and Mo_3_S_13_ cluster on the AlGaN (10$\bar 1$0). The hybrid functional was used to get more accurate results. (More calculated details could be seen in DFT calculation part). The density of states (DOS) of clean AlGaN and clean Mo_3_S_13_ are calculated using in hybrid functional is shown in Fig. 5c. Due to the influence of surface state and functional empirical parameters, there will be energy states in the middle of forbidden band of AlGaN surface. These results are similar to the previous works^68^. In Fig. S17, the DFT calculated bandgap of AlGaN alloy is ~4.15 eV, which is close to the bandgap value of 4.3 eV based on our PL measurement (The PL peak waveleng is 290 nm). Such small difference between them comes from the hybrid exchange-correlation functional used in our DFT calculations^69^. In fact, the stoichiometry of the AlGaN nanowire is around 36%. The extracted bandgap and the corresponding Al composition in the AlGaN alloy is consist with an earlier experimental report^70^. From Fig. 5c, it is shown that the conduction band minimum and the valence band maximum of clean AlGaN and clean Mo_3_S_13_ formed the staggered gap. Figure 5d shows the DOS of Mo_3_S_13_ clusters on the AlGaN (10$\bar 1$0), the bandgap remains staggered gap, and the Mo_3_S_13_/AlGaN belongs to type-II band alignment^71^, which is also been proved in Fig. S18. This band structure can reduce hole-electron recombination and promote the effective separation of photoexcited carriers. In essence, we believe that the decoration of an ultrathin Mo_3_S_13_ shell on nanowire drastically promotes the HER reaction efficiency with improved carrier separation characteristic that triggers the reversal of photocurrent direction.

## Discussion

Here, by integration of high-crystal-quality III-nitride nanowires with amorphous molybdenum sulfides (a-MoS_x_) to form a core-shell heterostructure nanowires as photoelectrodes, we demonstrate a photoelectrochemical photodetector exhibiting spectrally discriminative characteristics. Through controlled electrodeposition methods, a thin a-MoS_x_ shell is uniformly coated on the highly crystallized p-AlGaN/n-GaN nanowires. Large photoresponse with opposite polarities under 254/365 nm illumination are obtained, and their responsivity and photocurrent can be further varied by adjusting the surrounding environment (applied bias, electrolyte concentration, etc.). Furthermore, the DFT simulation results are in line with experiments, confirming the successful adsorption energy modification of the nanowires after a-MoS_x_ decoration. In essence, the a-MoS_x_@p-AlGaN/n-GaN nanowire-based photoelectrochemical device architecture demonstrated in this work provides a new route for multiple-band spectrally distinctive photodetection.

## Materials and methods

### The growth of nitride nanowires on Si substrates by molecular beam epitaxy

The AlGaN/GaN heterojunction nanowires were grown on an n-doped Si (111) substrate by plasma-assisted MBE techniques. The Si substrate was cleaned by the following two steps prior to nanowire growth. Step 1: the substrate was first cleaned by the acetone and methanol solutions under ultrasonic condition to remove the organic contaminants and then the substrate was dried with pure nitrogen gas. Step 2: the native silicon oxides on the Si substrate was removed by 10% hydrofluoric acid (HF). Then the substrate was loaded into the MBE chamber and the residual oxides were further desorbed by in situ annealing at 780 °C. During the growth, the Al, Ga, Si, and Mg fluxes were controlled using respective thermal effusion cells, whereas the nitrogen radicals were supplied from an RF-plasma source. The growth was conducted in nitrogen-rich conditions to form N-terminated surface to improve the stability of the nanowires in the PEC environment. The n-GaN segment was grown at 780 °C with Si doping and the p-AlGaN segment was grown at 870 °C with Mg doping. The resulting nanowires consist of ~200 nm n-GaN and ~200 nm p-AlGaN (on top of the n-GaN), with a total length of ~400 nm and a diameter of ~60 nm. The p-doping of AlGaN alloy is 5.6 × 10^16^ cm^−3^ and the n-doping of GaN alloy is 2.5 × 10^19^ cm^−3^, respectively.

### The fabrication and preparation of the photoelectrodes

We used the Ga-In eutectic alloy from Alfa Aesar as the metal contact to coated on the cleaned Si substrate from backside. Thereafter, the entire chip was loaded onto a cooper sheet by utilizing silver paste, and then encapsulated by insulating epoxy (except the area where the nanowires were grown for light exposure). Note: The photoelectrodes should be carefully covered by the insulating epoxy, otherwise, the photogenerated current might leak through the electrodes. Prior to any measurement, the photoelectrodes have to be dried in air for at least 24 h.

### Amorphous MoS_x_ decoration

The a-MoS_x_@III-Nitride nanowires were prepared by a facile electrodeposition method similar to previous report^19,28^. Before electrodeposition, the p-AlGaN/n-GaN nanowires photoelectrode was rinsed by distilled water for several times. Then, the electrode is immersed into (NH_4_)_2_MoS_4_ aqueous solution with a desired concentration. Using the saturated Ag/AgCl and Pt mesh as the reference and counter electrode in three-electrode configuration, 20 cycles of cyclic voltammetry were conducted, with a scan rate of 50 mV/s. After the electrodeposition, the MoS_x_-decorated p-AlGaN/n-GaN nanowires photoelectrode was washed with distilled water several times.

### Material characterizations

The morphologies of the nanowires were characterized by SEM on Hitachi, SU8220 systems. TEM, EDS elemental mapping were obtained on a 26FEI Talos F200X device at 200 kV. HAADF–STEM, high-resolution TEM results were obtained on a JEM-ARM 200 F instrument at 200 kV. XPS measurements were carried out on a Thermo Scientific K-Alpha XPS instrument equipped with an Al Kα source (*hν* = 1486.68 eV). The binding energies were calibrated by the reference of C 1s at 284.8 eV. The room-temperature PL signal was excited using a 266 nm pulsed laser source and collected through an ultraviolet objective by an OceanOptics QEPro spectrometer.

### Photoelectrochemical measurements

The photoelectrochemical performance of the spectrally distinctive photodetector was conducted on a CHI 660E electrochemical workstation with a typical three-electrode setup. The photoelectrochemical photodetector was constructed in a high-UV-transmittance quartz reaction cell. The saturated Ag/AgCl and Pt mesh electrode was used as the reference and counter electrode, respectively. The light source with wavelengths of 254 and 365 nm was obtained by a UV lamp, while the evolution of the normalized photocurrent was measured under LEDs with different wavelengths. The light intensities were calibrated by an optical power meter (Newport Model No. 2936 R). The photocurrent density *I*_photo_ was calculated as follows:

$$I_{{\rm{photo}}} = I_{{\rm{light}}} - I_{{\rm{dark}}}$$

where *I*_light_ and *I*_dark_ are the current density with or without light, respectively, as extracted from the *I*–*t* curves.

### DFT calculation

The DFT calculations have been performed by using the Vienna Ab initio Simulation Package^72,73^. The Generalized Gradient Approximation with the Perdew-Burke-Ernzerhof exchange-correlation functional^74^ was used to deal with exchange and correlation interactions of electrons. Pseudopotentials implemented in the Projector Augmented Wave method^75^ were used to model core electrons. The energy cutoff was adopted as 400 eV. The effect of the van der Waals interactions was considered by the DFT-D3 method proposed by Grimme et al.^76^. All atoms were allowed to relax until the forces were less than 0.02 eV Å^–1^ and total energies were converged to 10^−5^ eV. The Brillouin zones were sampled with a grid of 6 × 6 × 6 for the primitive cells of AlGaN bulk phase and 1 × 1 × 1 for other structures according to the Monkhorst-Pack procedure. The model of (10$\bar 1$0) surface of AlGaN was constructed with six Al-Ga-N layers, where the bottom three layers were fixed at the corresponding bulk structure. A vacuum layer which the thickness was 15 Å was inserted between the periodically repeated slabs along the c-axis to avoid interactions among them. The fraction of exact exchange in the hybrid functional calculation (AEXX = 0.33) was used to give a more reliable electronic structure.

In general, the ideal HER catalyst needs to meet the Gibbs free energy of hydrogen adsorption (Δ*G*_H_*) close to 0 eV. That is to say, under standard conditions, the HER pathway can be described by:

$${\rm{H}}^ + + {\rm{e}}^ - + {\rm{H}}^ \ast \to {\rm{H}}_2$$

where the H* represents a hydrogen atom adsorbed on the surface. The Gibbs free energy of the adsorbed state can be calculated by this formula^77^:

$$\Delta G_{{\rm{H}}^ \ast } = \Delta E_{{\rm{H}}^ \ast } + \Delta E_{{\rm{ZPE}}} - T\Delta S_{\rm{H}}$$

where $\Delta E_{{\rm{H}}^ \ast }$ is the hydrogen chemisorption energy, ∆*E*_ZPE_ and *T*∆*S*_H_ is the difference in zero point energy and entropy energy between the adsorbed and the gas phase. Each term can be calculated using standard thermodynamic methods. And the slab is fixed when we calculate the zero point energy, so we only calculate the zero point energy of adsorbed state and the hydrogen.

## Supplementary information

Supplementary information for publication
