# Supplementary material for: Observation of polarity-switchable photoconductivity in III-nitride/MoSx core-shell nanowires
Source: Light Sci Appl. 2022 Jul 19;11:227. doi: 10.1038/s41377-022-00912-7 (PMC9296537; doi:10.1038/s41377-022-00912-7)
Supplement: Supplementary file 1 — Supplementary information for publication [file 41377_2022_912_MOESM1_ESM.docx]

**Supplementary Information for**

**Observation of Polarity-Switchable P****hotoconductivity in III-nitride/MoS_x_ Core-Shell Nanowires**

*Danhao Wang^1,#^,* *Wentiao Wu*^2,^*^#^, Shi Fang^1,#^, Yang Kang^1^, Xiaoning Wang^2^, Wei Hu^2*^, Huabin Yu^1^, Haochen Zhang^1^, Xin Liu^1^, Yuanmin Luo^1^, Jr-Hau He^3^, Lan Fu^1,4^, Shibing Long^1^, Sheng Liu^5*^ and Haiding Sun^1,6*^*

^1^School of Microelectronics, University of Science and Technology of China, Hefei 230029, China

^2^Hefei National Laboratory for Physical Sciences at the Microscale, Department of Chemical Physics, University of Science and Technology of China, Hefei 230029, China

^3^Department of Materials Science and Engineering, City University of Hong Kong, Kowloon, Hong Kong SAR, 999077, China

^4^Department of Electronic Materials Engineering, Research School of Physics and Engineering, The Australian National University, Canberra, ACT 2601, Australia

^5^School of Microelectronics, Wuhan University, Wuhan 430072, China

^6^The CAS Key Laboratory of Wireless-Optical Communications, University of Science and Technology of China, Hefei 230029, China

*^#^*These authors contributed equally to this work.

Correspondence:

Haiding Sun: Email: [haiding@ustc.edu.cn](mailto:haiding@ustc.edu.cn)

Wei Hu: Email: [whuustc@ustc.edu.cn](mailto:whuustc@ustc.edu.cn)

Sheng Liu: Email: shengliu@whu.edu.cn

**Supplementary Figures**

**Fig. S1** Photoluminescence spectrum of the MBE-grown p-AlGaN/n-GaN nanowires. The peak at ~290 nm can be denoted as p-AlGaN segment, while peak around 365 nm comes from n-GaN segment.

**Fig. S2** Schematic illustration of 365 nm photodetection process of bare p-AlGaN/n-GaN nanowires.

**Fig. S3** Schematic illustration of the surface band bending of (a) p-AlGaN and (b) n-GaN segment after exposing to electrolyte. The open-circuit potential (OCP) measurements and their values of (c) p-AlGaN and (d) n-GaN nanowires under 254 nm light illumination.

When p-AlGaN surface is in contact with water-based electrolyte, the downward band bending appears at p-type semiconductor surface^1^. The downward band bending pushes the photogenerated electrons drift to the surface to participate the hydrogen evolution reaction^2,3^. Similarly, when the n-type GaN surface is immersed in water-based electrolyte, the upward band bending appears at n-type semiconductor surface^4^. The upward band bending forces the photogenerated holes drift to the surface to participate in the oxygen evolution reaction^5,6^.

The OCP values of both p-AlGaN and n-GaN nanowires changed with and without illumination, confirming the existence of surface band bending and indirectly identifying the doping type of the nanowires(for p-AlGaN material, the ΔOCP is positive; for n-GaN material, the ΔOCP is negative).

Scientists have successfully identified and quantified the surface band bending in nanowires through various approaches, including the electrochemical methods (OCP, Mott–Schottky)^7,8^, kelvin probe force microscopy (KPFM) based surface photovoltage spectroscopy (SPS)^9,10^, angle-resolved X-ray photoelectron spectroscopy (ARXPS) valence spectrum^1,4^, electron paramagnetic resonance (EPR) spectra^11^, in-situ electrical bias dependent PL, photocurrent, and spin trapping experiments^12-14^.

**Fig. S4** Schematic illustration of 254 nm photodetection process of bare p-AlGaN/n-GaN nanowires. The major charge transport processes are indicated by the solid arrows.

**Fig. S5** Schematic illustration of 254 nm photodetection process of amorphous MoS_x_ decorated p-AlGaN/n-GaN nanowires. The major charge transport processes are indicated by the solid arrows.

**Fig. S6** The cyclic voltammograms (CV) was recorded during the electrodeposition of the a-MoS_x_ shell. The scan was conducted from 0.1 V to -1.0 V with a scan rate of 50 mV/s. The black arrows indicate the scan directions and the blue arrow indicates the revolution of the CV curve with increasing cycles, which agrees well with previous reports^15,16^, suggesting the successful deposition of a-MoS_x_.

**Fig. S7** Low-magnification bright-field TEM image of nanowire arrays (scale bar = 100 nm).

**Fig. S8** STEM EDS elemental mapping of a-MoS_x_@p-AlGaN/n-GaN nanowires, including Mo, S, Al, Ga, and N elements.

**Fig. S9** (a) Schematic diagram and (b) optical image of the three-electrode configuration of our photoelectrochemical photodetector. (c) Schematic diagram of as fabricated and packaged photoelectrode.

**Fig. S10** Electrochemical impedance spectroscopy (EIS) analysis of the p-AlGaN/n-GaN and a-MoS_x_@p-AlGaN/n-GaN nanowires under dark conditions. The EIS diameter of the a-MoS_x_ decorated nanowires is much smaller than that of the pristine nanowires, suggesting that the a-MoS_x_ can significantly improve the charge transport properties at the nanowire/electrolyte interface.

**Fig. S11** The bias dependent photoresponse of a-MoS_x_@p-AlGaN/n-GaN under (a)254 nm and (b)365 nm illumination.

**Fig. S12** The response/recovery time of the a-MoS_x_@p-AlGaN/n-GaN nanowires under 365 nm irradiation in 0.5 M H_2_SO_4_ as a function of the applied voltage. All of the t_res_ and t_rec_ values under different external voltage still remained at ~ms level.

**Fig. S13** The response/recovery time of the a-MoS_x_@p-AlGaN/n-GaN nanowires as a function of the concentration of H^+^ under the illuminations of (a) 254 nm and (b) 365 nm light.

As we shown in Figure 4c, when the concentration of H_2_SO_4_ ranged from 0.001 to 0.5, the 254 nm photocurrents are negative and the response/recovery time remains at ~s level. When the electrolyte changed to Na_2_SO_4_ (in other words, the concentration of H_2_SO_4_ is 0), the photocurrent is positive with ~ms level response/recovery time. Interestingly, all response/recovery time of nanowires in different concentration of H_2_SO_4_ solutions under 365 nm remain at ~ms level and display as positive photocurrent. In Fig S13a, we notice that the response time is higher than the recovery time for 0.5M H_2_SO_4_ concentration. This can be explained as follows:

On one hand, under 254 nm irradiation, the response time nearly remains at a constant level (~0.35 s), which can be explained as follows: In the dark condition, the H^+^ ions nearby the nanowires are excessive, no matter what the concentrations of H_2_SO_4_ is. The response time represents how fast the photocurrent is generated when the light turns on. Once the photocarriers are generated, the electrons can immediately participate into HER. In another word, the key limiting factor for response time (t_res_) is the generation rate of photocarriers, not the H^+^ ions concentrations.

On the other hand, the recovery time decrease drastically when the concentration of H^+^ increases, as shown in Fig S13a. To reveal the factor that affects the recovery time (t_rec_) which represents the speed of photocurrent recovery once the light is turned off, we must understand that the recover speed also represents how fast the remaining photogenerated carriers inside nanowire drifted to the nanowire surface to participate into the chemical reactions. As we know, before the light is turned off, the H^+^ ions concentration nearby the nanowires is at a balance between the consumption by the HER process and the migration of ions due to the concentration gradients. Once the light is turned off, no more photocarriers are being generated, thus the balance is broken. As the concentration of H_2_SO_4_ increases, the conductivity of the electrolyte and the amounts of reagent increase, which could boost the consumption rate of photogenerated carriers, leading to a significant decrease of the recovery time (t_rec_). As a result, the response time is higher than the recovery time for 0.5 M H_2_SO_4_ concentration.

**Fig. S14** The photocurrent of (a) n-GaN nanowires and (b) p-AlGaN nanowires under 254 nm and 365 nm illumination.

Because n-GaN has a large absorption coefficient when it is illuminated by 254 nm compared with 365 nm light^17^, the photocurrent of n-GaN under 254 nm is larger than photocurrent of n-GaN under 365 nm. The solar-blind/UV rejection ratio (R254 nm/R365 nm) of pure n-GaN nanowires is 4.15. The UV/solar-blind rejection ratio (R365 nm/R254 nm) of pure p-AlGaN nanowires is 0.003.

**Fig. S15** The SEM images of a-MoSx@p-AlGaN/n-GaN nanowires (a-b) before and (c-d) after PEC test in acid environment.

**Fig. S16** The calculated free energy profiles of the OER steps on Mo_6_S_24_ (blue line) and AlGaN (10$\bar{1}$0) (red line) at potential of 1.23 V versus Standard Hydrogen Electrode (SHE). The OER process mainly involves four-electron process, which occur through the following steps:

$$*+H_{2}O\to*OH+H^{+}+e^{-}$$

$$\boldsymbol{*}OH\to*O+H^{+}+e^{-}$$

$$\boldsymbol{*}O+H_{2}O\to\boldsymbol{*}OOH+H^{+}+e^{-}$$

$$\boldsymbol{*}OOH\to*+O_{2}+H^{+}+e^{-}$$

The associated Gibbs free adsorption energies $\Delta G$ in each step are expressed as follows:

$$\Delta G=\Delta E+{\Delta E}_{ZPE}-T\Delta S+{\Delta G}_{U}+{\Delta G}_{PH}$$

where $\Delta E$ is the total energy difference between reactants and products of reactions, and${\Delta G}_{U}=-eU$,$eU$ is the shift in electron energy with applied bias, ${\Delta G}_{PH}$ is the correction of the $H^{+}$ free energy, the $\Delta G$ of four process is remark as ${\Delta G}_{1}$, ${\Delta G}_{2}$, ${\Delta G}_{3}$,${\Delta G}_{4}$.

For an ideal catalyst, the Gibbs free energy for each elementary step is the same^18^: ${\Delta G}_{1}={\Delta G}_{2}={\Delta G}_{3}={\Delta G}_{4}$. For actual catalysts, the G for these four items aren’t equal. The over potential η can be determined by the largest Gibbs free energy differences of each step as:

$$\eta=max[({\Delta G}_{1},{\Delta G}_{2},{\Delta G}_{3},{\Delta G}_{4})/e]$$

The step of over potential η are also called by the rate-determining step (RDS). Obviously, both Mo_6_S_24_ and AlGaN (10$\bar{1}$0), the RDS is the formation of OOH* from O* with overpotential which Mo_6_S_24_ is 0.62V and AlGaN (10$\bar{1}$0) is 0.90V. The higher reaction overpotential of AlGaN (10$\bar{1}$0) than Mo_6_S_24_, suggest that the chemical adsorption of the intermediates on AlGaN (10$\bar{1}$0) is very strong, leading to a high activation barrier for the OER to proceed, which is consists well with our photodetection performance results (Figure 3c)

**Fig. S17** (a) The atomic configuration of bulk AlGaN, the balls in light blue, navy and pink represents N, Al and Ga atoms, respectively. (b) Calculated density of states of bulk AlGaN.


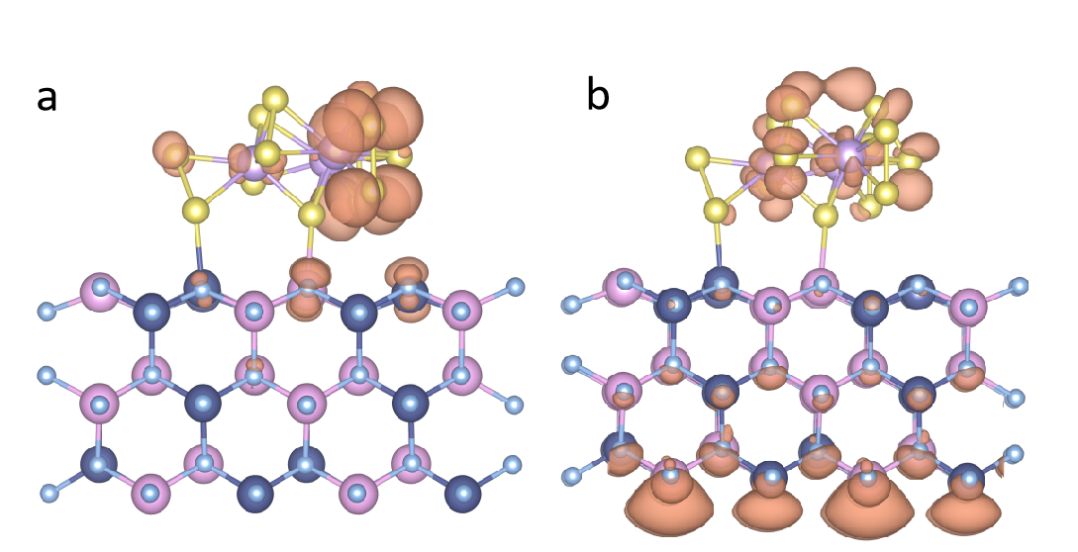


**Fig. S18** Charge density distribution of (a) VBM and (b) CBM. The balls in yellow, purple, light blue, navy and pink represent S, Mo, N, Al and Ga atoms, respectively.

To further support our conclusions, we plotted the charge densities of CBM and VBM using the band-decomposition charge densities. As far as we know, the staggered type heterostructure facilitates carrier separation, in other word, can reduce the recombination of electron-hole pairs^19^. In our calculation model, the majority of electrons in VBM are concentrated on Mo_3_S_13_, whereas in CBM they are concentrated in the deep layer of AlGaN. These separated CBM and VBM bands also show that the Mo_3_S_13_/AlGaN interface is a staggered type.

**References:**

1. Kibria, M. G. *et al.* Tuning the surface Fermi level on *p*-type gallium nitride nanowires for efficient overall water splitting. *Nature Communications* **5**, 3825 (2014).

2. Wang, D. H. *et al.* Pt/AlGaN nanoarchitecture: toward high responsivity, self-powered ultraviolet-sensitive photodetection. *Nano Letters* **21**, 120-129 (2021).

3. Kamimura, J. *et al.* *p*-type doping of GaN nanowires characterized by photoelectrochemical measurements. *Nano Letters* **17**, 1529-1537 (2017).

4. Kibria, M. G. *et al.* Visible light-driven efficient overall water splitting using *p*-type metal-nitride nanowire arrays. *Nature Communications* **6**, 6797 (2015).

5. Wang, D. H. et al. Highly uniform, self‐assembled AlGaN nanowires for self‐powered solar‐blind photodetector with fast‐response speed and high responsivity. *Advanced Optical Materials* **9**, 2000893 (2021).

6. Wang, D. F. *et al.* Wafer-level photocatalytic water splitting on GaN nanowire arrays grown by molecular beam epitaxy. *Nano Letters* **11**, 2353-2357 (2011).

7. Kornienko, N. *et al*. Growth and photoelectrochemical energy conversion of wurtzite indium phosphide nanowire arrays. *ACS nano* **10**, 5525-5535 (2016).

8. Zhao, C. *et al*. Quantified hole concentration in AlGaN nanowires for high-performance ultraviolet emitters. *Nanoscale* **10**, 15980-15988 (2018).

9. Li, Z. et al. Surface‐polarit‐induced spatial charge separation boosts photocatalytic overall water splitting on GaN nanorod arrays. *Angewandte Chemie International Edition* **132**, 945-952 (2020).

10. Doughty, R. M. *et al.* Surface photovoltage spectroscopy observes junctions and carrier separation in gallium nitride nanowire arrays for overall water-splitting. *The Journal of Chemical Physics* **153**, 144707 (2020).

11. Liu, M. X. *et al.* GaN nanowires as a reusable photoredox catalyst for radical coupling of carbonyl under blacklight irradiation. *Chemical Science* **11**, 7864-7870 (2020).

12. Philipps, J. M. *et al.* Photoelectrochemical response of GaN, InGaN, and GaNP nanowire ensembles. *Journal of Applied Physics* **123**, 175703 (2018).

13. Kamimura, J. *et al.* Broad band light absorption and high photocurrent of (In, Ga) N nanowire photoanodes resulting from a radial stark effect. *ACS Applied Materials & Interfaces* **8**, 34490-34496 (2016).

14. Lähnemann, J. *et al.* Radial stark effect in (In, Ga) N nanowires. *Nano Letters* **16**, 917-925 (2016).

15. Merki, D., Fierro, S., Vrubel, H. & Hu, X. L. Amorphous molybdenum sulfide films as catalysts for electrochemical hydrogen production in water. *Chemical Science* **2**, 1262-1267 (2011).

16. Vrubel, H. & Hu, X. L. Growth and activation of an amorphous molybdenum sulfide hydrogen evolving catalyst. *ACS Catalysis* **3**, 2002-2011 (2013).

17. Xia, S. H. *et al.* Research on quantum efficiency of GaN wire photocathode. *Optical Materials* **64**, 187-192 (2017).

18. Feng, C. *et al.* Fe-based electrocatalysts for oxygen evolution reaction: progress and perspectives. *ACS Catalysis* **10**, 4019-4047 (2020).

19. Marschall R. Semiconductor composites: strategies for enhancing charge carrier separation to improve photocatalytic activity. *Advanced Functional Materials* **24**, 2421-2440 (2014).
